# Mapping between sound, brain and behaviour: four-level framework for understanding rhythm processing in humans and non-human primates

**DOI:** 10.1098/rstb.2020.0325

**Published:** 2021-10-11

**Authors:** Tomas Lenc, Hugo Merchant, Peter E. Keller, Henkjan Honing, Manuel Varlet, Sylvie Nozaradan

**Affiliations:** ^1^ The MARCS Institute for Brain, Behaviour and Development, Western Sydney University, Penrith, New South Wales 2751, Australia; ^2^ School of Psychology, Western Sydney University, Penrith, New South Wales 2751, Australia; ^3^ Instituto de Neurobiologia, UNAM, Campus Juriquilla, Querétaro 76230, Mexico; ^4^ Amsterdam Brain and Cognition (ABC), Institute for Logic, Language and Computation (ILLC), University of Amsterdam, Amsterdam 1090 GE, The Netherlands; ^5^ Institute of Neuroscience (IONS), Université Catholique de Louvain (UCL), Brussels 1200, Belgium

**Keywords:** rhythm, musical meter, mapping, transformation, frequency-tagging, neurophysiology

## Abstract

Humans perceive and spontaneously move to one or several levels of periodic pulses (a meter, for short) when listening to musical rhythm, even when the sensory input does not provide prominent periodic cues to their temporal location. Here, we review a multi-levelled framework to understanding how external rhythmic inputs are mapped onto internally represented metric pulses. This mapping is studied using an approach to quantify and directly compare representations of metric pulses in signals corresponding to sensory inputs, neural activity and behaviour (typically body movement). Based on this approach, recent empirical evidence can be drawn together into a conceptual framework that unpacks the phenomenon of meter into four levels. Each level highlights specific functional processes that critically enable and shape the mapping from sensory input to internal meter. We discuss the nature, constraints and neural substrates of these processes, starting with fundamental mechanisms investigated in macaque monkeys that enable basic forms of mapping between simple rhythmic stimuli and internally represented metric pulse. We propose that human evolution has gradually built a robust and flexible system upon these fundamental processes, allowing more complex levels of mapping to emerge in musical behaviours. This approach opens promising avenues to understand the many facets of rhythmic behaviours across individuals and species.

This article is part of the theme issue ‘Synchrony and rhythm interaction: from the brain to behavioural ecology’.

## 1. Meter is fundamental for temporal coordination

Humans across cultures engage in collective musical behaviours such as ensemble performance and dance, which involve precise temporal coordination of actions between individuals [1,2]. In such scenarios, information used for coordination is delivered through dynamically changing acoustic (as well as visual and tactile) inputs that stimulate the sensory organs. Importantly, both the auditory input and behavioural output can take the form of non-periodic (i.e. non-repeating), mutually distinct sequences, which supports the emergence of complex creative behaviours. In a string quartet, for example, each musician produces a complex series of movements that may not systematically repeat (i.e. are not necessarily periodic). Still, these movements are precisely coordinated in time with the acoustic input the musician receives, which comprises a complex (again, not necessarily periodic) aggregate sound sequence of the whole ensemble. A powerful way to coordinate such signals involves using an internal temporal reference that is largely invariant to the particular pattern of dynamic changes in the sensory input, yet can be shared and synchronized across individuals.

A fundamental form of temporal reference is based on an internal representation of a pulse, i.e. a series of regularly recurring (periodic) points in time [3,4]. In humans, such an internal representation of pulse is commonly established when listening to rhythmic stimuli ranging from strictly periodic metronomes to complex musical performances [5–7]. Moreover, humans often simultaneously represent multiple pulses with different periods arranged in a nested set [8–11], a phenomenon referred to as meter perception [6,7,12,13] (electronic supplementary material, figure S1). This nested set of metric pulses thus represents an internal temporal reference that can be used by a number of processes including movement planning [8,11,14–17], dynamic attention [18–22], anticipation of features of the sensory input [23,24] or encoding of time intervals making up the rhythmic input [25,26].

Traditionally, different terms were used to refer to one periodic level within the perceived metric set as the beat (or *tactus* in music theory [27]), and the other, slower and faster pulses, as grouping or subdivision of the beat periodic level [6,7,28]. Relatedly, meter is often described as a hierarchical structure, where the beat serves as a central time reference. However, as an operational definition for the purpose of the current review, the term ‘metric pulse’ will be used to refer to *any* constituent periodic level within a meter. In other words, we acknowledge that the number of periodic levels (i.e. pulses) in the perceived meter can differ across individuals and contexts (and perhaps in certain cases, only one periodic level can be perceived). But here this will be conceptualized as a quantitative (one, two or *N* periodic levels) rather than qualitative (categorizing pulses as beat, grouping, subdivision) distinction. Such an approach does not exclude the possibility of hierarchical organization in the way metric pulses are internally represented, but rather reduces the number of assumptions to allow for the development of a more general framework.

**Figure 1.**
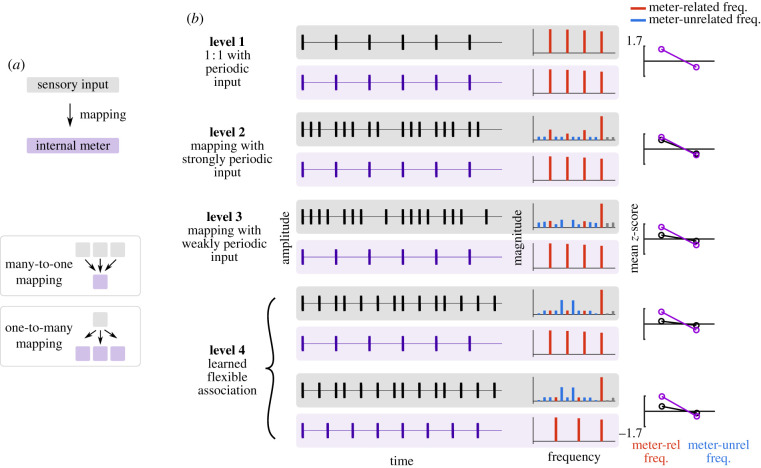
(*a*) Schematic of meter perception, which is a critical ability allowing temporal coordination in musical behaviours. The physical sensory input is mapped onto internal representations of time in the form of metric pulses. Different inputs can be mapped onto the same internal meter (many-to-one mapping) and the same input can be mapped onto different internal meters (one-to-many mapping). (*b*) Schematic of the framework decomposing meter processing into four levels. Each level is described with an example mapping from the sensory input (grey boxes) to internal meter (purple boxes) (here one pulse is chosen for simplicity). Note that the shape of the signals is chosen for illustrative purposes. The frequency-domain representations of the example signals show frequencies related to the internal metric pulse in red, and a set of meter-unrelated frequencies (yet prominent in the input spectra for levels 2, 3 and 4). Relative prominence of meter-related and -unrelated frequencies in the spectra was quantified using *z*-score normalization (note the enhancement of meter-related frequencies in signals indicating the internal representation). The chosen metric pulse is different for the two examples in level 4, while the input is identical, thus indicating one-to-many mapping.

It is important to stress that in musical behaviours, the mapping between internally represented metric pulses and the rhythmic modulations of physical signals is typically not one-to-one (figure 1*a*; electronic supplementary material, figure S1) [6,7,29]. Rather, different meters (i.e. with different periods and phase) can be perceived with the same rhythm, and the same meter (i.e. characterized by the same periods and phase) can be perceived with different rhythms. An example of such many-to-one mapping is when listeners perceive the same meter across different rhythms played by a musician at different time points during a performance.^1^ Another example of many-to-one mapping is when dancing a waltz (requiring representation of a meter where pulse periods have 1 : 3 relationship) to music as acoustically distinct as Strauss' The Blue Danube and Metallica's Nothing Else Matters. At the same time, physically identical stimuli can induce representation of different meters across individuals or contexts [15,31–33], which can be described as a form of one-to-many mapping.

In the last decades, an important aim of psychology and cognitive neuroscience has been to describe the nature of the processes that enable and shape the mapping between rhythmic sensory inputs and internal representation of metric pulses, to uncover their neural basis [34–36], how they develop over the lifetime [37–39] and to what extent they are present in other species [40,41]. To this aim, it is critical to use a valid and comparable method to measure the internal representation of metric pulses across these different contexts.

## 2. Approaches to measure internal representation of meter

Similar to other perceptual phenomena, the internal representation of meter cannot be measured directly in individuals. Instead, a variety of methods have been developed to infer this representation indirectly, based on a range of assumptions. A particularly fruitful family of methods has been based on analysing dynamic fluctuations of behavioural and neural signals in real time, as the individual is stimulated with rhythmic sensory inputs.

### (a) Approaches based on measuring secondary processes

One particularly influential approach to estimate the internal representation of meter is based on measuring the dynamics of a secondary perceptual or cognitive process. This approach is equivalent to probing whether (and how) the brain represents, for example, time, space or semantic content, by quantifying whether the system can use these dimensions to guide processes such as overt movement, attentional selection or expectations. Using such an approach to probe the internal representation of meter rests on three critical assumptions: (i) that the secondary process dynamically changes over time, (ii) these dynamic changes can be measured with appropriate temporal resolution, and (iii) these dynamic changes are coupled one-to-one with the internally represented metric pulses.

A commonly used process that may meet these assumptions is overt body movement [11,12,42]. This is owing to the intrinsic connection between meter and movement [43–46] in musical contexts across cultures [47], and the ability to directly measure movement dynamics in real time using motion capture equipment. Crucially, the assumption about one-to-one coupling between movement and internally represented metric pulse can be satisfied via instructions [15,16]. In other words, it is straightforward to ask human participants to directly move along with a periodic pulse they spontaneously perceive when listening to the rhythmic input (note that this is not conventional for many musical styles, e.g. in swing or Second Line music, enculturated listeners commonly move in antiphase with one of the perceived metric pulses by clapping or snapping fingers at mid-points between successive pulse positions). However, such instructions cannot be given to infants or non-human animals, where researchers must rely on one-to-one coupling of movement and internal metric pulses emerging spontaneously [37,42], an assumption that may depend on the nature of the sensory input [48]. Moreover, it is important to keep in mind that biomechanical constraints which limit movement at fast rates may not directly apply to the internal pulse representation. This is evidenced by the ability to internally represent faster pulses that can be directly executed through stable movement [49]. Yet, such considerations do not contradict the wide range of evidence suggesting that the internal representation of a metric pulse might be implemented within neural circuits involved in motor control [40,50]. Rather, the aim is to point out that in certain contexts, the actual executed movement sequences might not match this internal representation one-to-one, and that this possibility should be considered when using movement to infer internal meter representation.

Besides movement, it has been shown that several other psychological processes may use the internal representation of meter as a temporal reference. These include dynamic fluctuations of attentional sensitivity or dynamic attention [22], and predictions of the upcoming sensory input (‘when’ and ‘what’ predictions) [51]. Both, dynamic attention and prediction, can be indirectly measured through their behavioural or neural correlates. Measuring these correlates typically involves capturing responses to transient events in the sensory input. Depending on the targeted process, events may be defined as simple sounds [52–55], or sounds that violate a statistical rule established by preceding context (e.g. having greater or smaller intensity relative to the context) [9,24,56], or silences that violate a repeating sequential pattern [22,57,58]. If the targeted secondary process dynamically changes over time and is coupled one-to-one with the internal metric pulse (assumptions 1 and 3 listed above), the behavioural or neural response to a physically identical event should be consistently different depending on the temporal position of the event with respect to this internally represented metric pulse. At the behavioural level, the responses are typically measured in terms of perceptual sensitivity and reaction times. At the neural level, the responses can be captured as event-related potentials (time-locked fluctuations of field potentials elicited by the event; for a review, see [59]). Capturing these processes through their neural correlates is advantageous, as responses from infants [58] or non-human animals [60] can be measured. However, similar to movement, the assumption about one-to-one coupling between these processes and internally represented metric pulses must be carefully considered before using them to make inferences about the represented meter. While there is a considerable amount of evidence suggesting that fluctuations of attention and prediction can be directly coupled to the metric pulses [18,19,61,62], some studies indicate that this may depend on stimulus properties, behavioural goals and prior experience of the individual [23,63–65]. Moreover, unlike movement, these processes may be harder to control via explicit instructions.

If the assumptions are carefully considered, measuring processes such as overt movement or dynamic attention constitutes a powerful tool to estimate the internal representation of metric pulses.

### (b) Approaches based on measuring neural representations

Instead of measuring a secondary process that uses the internal representation of metric pulses as a time reference, an alternative approach aims to directly capture the neural representation of metric pulses. Hence, this approach does not rely on the assumption about the coupling of a secondary process with the internally represented metric pulses. Rather, it is based on the assumption that the internal representation of a pulse is encoded in the dynamic fluctuations of neural activity that can be measured using neuroscientific methods [34,66–69]. The fact that neural activity can be captured at multiple spatial scales, and in a number of ways (e.g. as single-unit firing rates, population trajectories, amplitude modulations of band-limited local field potentials or large-scale electroencephalogram (EEG) activity), may provide complementary insights into the way metric pulses are represented in the nervous system, and into the nature of the transformation processes that support the mapping between internal representations of metric pulses and the sensory input [29,46,67,70–72].

Using this approach is valid even for neural signals of complex physiological origin (such as EEG) that probably reflect a mixture of lower- and higher-level processes related to the internal representation of the sensory input [29,73,74]. While such signals cannot be expected to directly isolate neural representation of metric pulses, they can be assumed to be systematically influenced by this representation. Hence, in carefully designed experiments and analysis methods, such responses can provide valuable insights into the nature of internal meter representation. It is crucial to note that large-scale neural responses such as field potentials can hardly reveal low-level neurophysiological implementation of metric pulse processing (this can be only achieved by directly capturing neuronal firing rates or layer-specific dendridic currents, e.g. [75,76]). Rather, these neural signals can be used as a powerful complement to behavioural responses (offering several advantages owing to little reliance on decisional processes and movement abilities). Moreover, when recorded intracranially, field potentials can offer critical insights into the spatio-temporal characteristics of the neural network involved in meter processing ([72,77], see also [78]).

### (c) Quantifying pulse prominence

Once a behavioural or neural signal has been measured while the individual was stimulated with a particular rhythm, the goal is now to measure how prominent a particular set of metric pulses is within the captured signal. To this goal, a range of analysis methods can be used, and these different analytic tools are linked to a common principle which is based on the definition of pulse, i.e. *periodic recurrence*. Particularly, periodic recurrence can be decomposed into three crucial components (see the electronic supplementary material, figure S2) that will all need to be quantified in the signal: (i) a part of signal's dynamic trajectory in the state space must consistently repeat (self-similarity), (ii) this repetition must occur precisely separated by the time interval given by the pulse period (regularity), and (iii) the trajectory must be otherwise different from the repeated part (contrast). Here, the state space is a general term that captures possible patterns of features relevant for the signal (e.g. amplitudes measured at different sensors).

It is important to note that periodic recurrence can be considered a quantitative property. That is, a signal characterized by a greater degree of self-similarity, contrast and regularity at a particular period may be considered to comprise a more prominent representation of a pulse with that period. Moreover, because a variety of signals can show comparable self-similarity, contrast and regularity, it follows that equivalent pulse representation might be implemented in signals with various shapes and formats (see the electronic supplementary material, figure S3). Hence, a valid method to quantify the prominence of a pulse would generalize across signal properties irrelevant to periodic recurrence, but remain sensitive to self-similarity, contrast and regularity. To this aim, several families of measures have been developed.

Firstly, methods based on phase analysis have been proposed [12]. These phase-based methods are sensitive to the regularity of signal trajectory with respect to pulse period. However, they are less sensitive to the contrast property (see the electronic supplementary material, figure S4 for an example). Secondly, contrast-based methods assume that periodicity in the signal is driven by transient features (such as high-amplitude bursts) that differ within narrow time windows centred on- versus off- pulse positions [74,79,80]. However, these measures might be less sensitive to fine temporal regularity (see the electronic supplementary material, figure S4). Finally, a third family of measures include autocorrelation (based on high self-similarity of a periodic signal when shifted by time equivalent to the pulse period) ([72]; see the electronic supplementary material, figures S4 and S5) and frequency-tagging (based on the fact that periodic recurrence in a signal can be identified in the frequency domain as narrow-band peaks of energy centred at the frequencies corresponding to the pulse period and its harmonics) [29,81].

These different methods and their respective assumptions, advantages and pitfalls are illustrated and further discussed in the electronic supplementary material, figure S4. In order to make valid conclusions about pulse prominence, it is crucial to consider these strengths and weaknesses, as well as the nature of the analysed signals. In many cases, the different measures might be used in a complementary way to provide better insight into pulse representation within a signal (e.g. [82,83]).

### (d) Taking into account pulse prominence in the physical input

In order to investigate the nature of the mapping between the sensory input and internal metric pulses, the physical properties of the sensory input itself must be taken into account. How prominent a pulse is within the physical input has been estimated, for example, using methods based on contrast [4,79,82,84], frequency-tagging [77,81,83,85,86] or autocorrelation [72,87]. Investigating the sensitivity of the perceptual system to the periodicity of various input features has contributed to understanding the mapping from rhythmic input to internal meter [4,74,88,89], and has led to the development of extensive models of pulse and meter perception [46,88,90,91]. However, it is important to note that the aims of analysing pulse prominence in the physical input also go beyond providing a comprehensive model of perceptual experience. In fact, analysing the physical stimulus is critical to capture the relevant transformation underlying the mapping from rhythmic input to internal meter. The term ‘transformation’ is thus used here to emphasize the range of neural processes from the peripheral receptors to cortical pathways, which are involved in the mapping from physical input to meter by gradually transforming the internal representation from faithful tracking of input physical features towards higher-level behaviourally relevant categories (metric pulse(s) in our case) [29,92–94].

There is converging evidence that the brain can faithfully track modulations of various input features [95–97]. Hence, observing a prominent periodicity in behavioural or neural responses may be trivially explained if this periodicity is already prominent in these physical features [91]. Therefore, quantifying the prominence of periodicities in the acoustic input is critical to avoid such trivial conclusions [92,98]. In the case of metric pulses, this can be done by directly comparing the prominence of metric pulses in signals representing a chosen input feature (e.g. amplitude envelope) and the elicited response (e.g. EEG activity, or finger tapping). This is valid if the measure under comparison is sensitive to signal properties that are relevant to the functional definition of the phenomenon (here the three properties making up periodic recurrence: self-similarity, contrast and regularity), while generalizing across units, scales and different possible representation formats [99]. As discussed in the electronic supplementary material, if used carefully, measures of pulse prominence based on frequency domain may fulfil these criteria [72,100]. Moreover, these methods can be used to make valid comparisons between signals captured from different brain regions at different spatial scales (including neuronal firing rates, local field potentials, narrow-band power fluctuations), as well as overt behaviour (e.g. finger tapping) [29].

In the following section, we discuss recent evidence based on capturing and directly comparing prominence of metric pulses in sensory inputs, elicited neural activity and behavioural outputs. Together, this evidence suggests that depending on input properties and context, the prominence of particular metric pulses may be gradually enhanced throughout the auditory pathway and the dorsal processing stream linking temporal, parietal and premotor cortices, thus providing a neural basis for mapping between external rhythmic signals and internal periodic pulse representations.

## 3. A range of processes involved in mapping from sensory input to metric pulses

The mapping from sensory input to internally represented meter has been often treated as a general ability. In other words, it is common to ask whether, for instance, non-human animals or human newborns can perceive the pulse (or a set of metric pulses) when listening to music [40,41,58]. However, there is increasing evidence that meter perception may be conceptualized as multiple processes which may be engaged differently depending on the nature of the sensory input and context [60,81,83,101]. These different processes can be described in terms of *what* the perceptual system does to map a sensory input onto an internal meter, and can be organized within four levels in which internal metric pulses are mapped (i) one-to-one with periodic input (level 1), (ii) with strongly periodic input (level 2), (iii) with weakly periodic input (level 3), and (iv) based on learned flexible associations (level 4). It is important to note that the boundaries between these proposed four levels are not categorical, but the processes involved at different levels might be involved in a graded fashion depending on the stimulus and context. Still, this four-level structure may constitute an insightful way to organize the growing and diverse body of empirical evidence on rhythm processing into a coherent framework, and guide future research. For example, the nature of the constituent processes making up each level remains to be identified, together with their specific constraints that may shape the mapping from specific inputs to specific internal meters. Moreover, the neural substrates underlying these constituent processes, how these processes differ between individuals and across species, and develop over the lifetime, remain largely unknown.

Finally, this conceptual four-level framework is generally compatible with influential theories, such as the neural resonance theory (NRT) [28,91], the sensory-motor theory [46,102] or predictive coding [51], which propose computational models to explain the processes highlighted across the four levels. Thus, the current aim is not to propose an alternative to these models. Rather, the goal of the proposed framework is to organize the empirical data these theories need to account for and to highlight the diversity of functional processes underlying rhythmic behaviour across individuals and species. Similar multi-level conceptual frameworks have stimulated progress in understanding phenomena such as theory of mind [103] or language [104].

### (a) Level 1: one-to-one mapping internal metric pulse with periodic input

At the most fundamental level, the metric pulse is directly represented already in the sensory input (as illustrated in figure 1*b*). This includes notionally isochronous, metronomic stimuli that contain periodic modulations of a physical feature. One-to-one mapping from such inputs onto internally represented pulses can be considered a fundamental ability for meter processing and has been observed across a number of species, including humans [12,49], but also insects [105].

**Figure 2.**
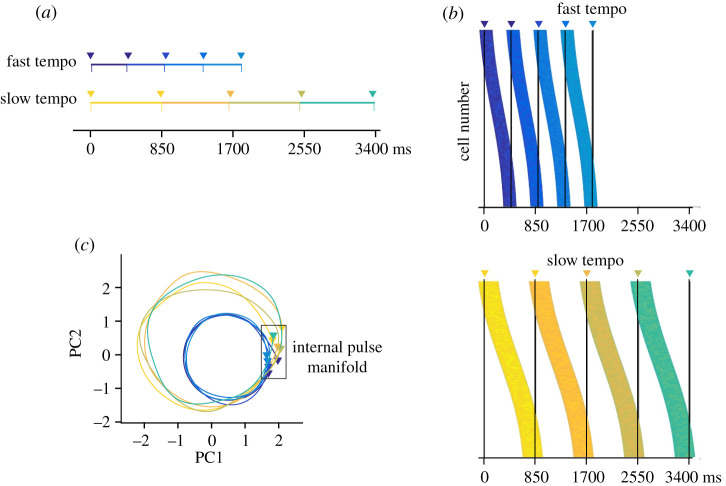
Neural population representation of an internal regular pulse during a tapping synchronization task. (*a*) Synchronization task. Monkeys are required to tap (triangles) synchronously with four brief pacing events (colour coded) of an external isochronous metronome (vertical lines). The stimulus onset asynchrony (SOA) creates fast and slow tempos. (*b*) Progressive neural activation patterns for fast (top) and slow (bottom) tempos. Each coloured strip consists of multiple horizontal lines, where each line corresponds to the onset and duration of the activation period for one cell. The simulated 200 cells are sorted by their peak activation time, generating for each produced interval an evolving population pattern of activation. The vertical lines correspond to the tapping times (triangles on top). Note that a similar population response profile is repeated in a cyclical manner for the four intervals (colour coded) and that the resetting of each moving bump corresponds to a potential internal pulse representation. (*c*) Neural trajectories during the synchronization task. The trajectory starts from the first tap, completes a cycle during every intertap interval and returns to the tapping manifold. We assume that the tapping attractor area in state space, highlighted by a box, is the internal pulse manifold which is invariant across durations. The metronome's tempo modulates the amplitude of the trajectories.

Recent findings also demonstrate that macaque monkeys have the neural machinery to map a metronomic (auditory or visual) stimulus onto an internal pulse representation, and coordinate tapping one-to-one with this pulse (figure 2*a*). Macaques show proper period matching, switching flexibly their tapping rate across a wide range of stimulus periods (450–1000 ms) [106]. Critically, they are capable of precisely timing their taps, such that they consistently occur slightly before the individual brief stimuli (e.g. tones or visual flashes) making up the periodic pacing sequence [107]. The ability to produce negative tapping asynchrony provides strong evidence that macaques do not simply react to the stimuli, but rather internally represent input period and use this internal representation to precisely time their movement. A similar predictive behaviour has been observed when monkeys were trained to synchronize their saccadic movements [108] or to internally calculate the position of a stimulus within a sequence of isochronously presented visual stimuli [109,110].

These observations support the view that monkeys have a sensory-motor system with all the anatomical and neurophysiological properties to compute the fundamental processing steps for one-to-one temporal coordination with a periodic stimulus [111]. Firstly, they must be able to extract the periodicity from a dynamically changing feature of the input. Hence, the characteristics of the relevant sensory system may constrain the ability to extract periodicity from inputs within specific modalities and limit the extraction process to specific input features [112]. For instance, the spontaneous preference to coordinate tapping with visual compared to auditory metronomic inputs observed in monkeys [106,107] might be partially driven by the lower sensitivity of their auditory system to low-frequency temporal modulations in acoustic inputs [113,114]. However, the auditory cortex of monkeys is still capable of spontaneously extracting the regularity of isochronous acoustic sequences, as evidenced by neural responses to sound omissions in an auditory metronome captured by scalp EEG on macaques [115]. Moreover, the fact that macaques show human-like frequency-following EEG responses elicited by fast acoustic periodicities suggests that their auditory system can precisely encode fine sound timing, thus providing a critical basis for coordination of movement with incoming acoustic inputs [116]. Secondly, the periodicity extracted from the sensory input must be mapped onto an internal pulse-like representation of time. Converging evidence suggests that the core timing network comprised by the cortico-thalamic-basal ganglia skeletomotor circuit (CTBGc) constitutes the neural underpinning of an internal pulse representation [117–119]. Specifically, the medial premotor cortex (MPC), composed of the supplementary motor cortex proper and the presupplementary motor area, is a key area for the conformation of an internal pulse signal [71]. Although the neural basis of these processing steps is still fairly unknown, the Rhesus monkey is a great model to study the dynamical interplay between bottom-up and top-down signals, using the simultaneous high-density single-cell recordings at different node levels of the beat-based timing audiomotor circuit [75]. In fact, critical observations have already been made in the primate MPC regarding the representation of a metric pulse during rhythmic tapping.

A key property of MPC neurons during rhythmic tapping to a metronome is the relative representation of pulse timing. Cells that encode elapsed or remaining time for a tap show up–down ramping profiles that span the produced time interval, scaling in speed as a function of pulse period [120,121]. Interestingly, neurons encoding elapsed or remaining time are tuned to the tapping rate, generating period-dependent subpopulations for pulse processing within MPC [122,123]. These neural signals are far from static. MPC cells are recruited in rapid succession producing a progressive neural pattern of activation (called moving bumps) that flexibly fills the time interval corresponding to one pulse period (figure 2*b*). This neural pattern may thus provide a relative representation of how far an interval between two successive metronome events has evolved [124,125]. Another critical aspect of the MPC periodic clock is that it resets on every pulse period cycle. Thus, the progressive pattern of activation starts with a group of cells, migrates to other cells during the timed interval, stops with the last group of cells and simultaneously is initialized for the next produced interval with the previous initial set of cells [70,125]. The neural cyclic evolution and resetting are more evident when the time-varying activity of MPC neurons is projected into a low-dimensional state space (figure 2*c*) [70]. The population neural trajectories show the following properties. First, they have circular dynamics that form a regenerating loop for every produced interval. Second, the periodic trajectories increase in amplitude as a function of the input period. These period-dependent increments in the trajectory radius are the result of a larger number of neurons within a moving bump [70]. Finally, the population neural trajectories converge in similar state space at tapping times, resetting the pulse-based clock at this point [70]. Hence, the convergence to this neural attractor state could be the internal representation of the pulse that is transmitted as a phasic top-down predictive signal to the auditory areas before each tap [70,126]. In addition, the population state during pulse-based predictive timing indicates the traversed proportion of an interval (relative timing) instead of its absolute magnitude. Consequently, these population dynamics can be the fundamental primordium of an internal signal of meter in humans, generating a predictive, relative-timing and flexible pulse signal within the MPC [36,126].

These findings are compatible with recent observations that low-dimensional population trajectories constitute a general mechanism to encode time in primate frontal cortices [127]. Yet, the establishment of an internal pulse representation is linked to a specific type of dynamics in the form of regenerating loops, which is distinct from the activity that emerges during interval reproduction tasks where the speed of dynamics is adjusted according to the timed interval [128,129]. This showcases how frontal motor regions can encode time predictively with a great deal of flexibility, thus supporting adaptive behaviour in a context-sensitive way.

#### (i) Adaptation and anticipation mechanisms

In the real world, rhythmic inputs that allow one-to-one mapping of a metric pulse are not perfectly periodic. Rather, ecologically valid stimuli often contain small variations in time-locking to a strictly isochronous pulse period, or tempo drift (i.e. gradual systematic change of the pulse period delivered by the input). Hence, real-time mechanisms must be used to maintain the precise one-to-one temporal coordination between the input and internal pulse representation (and consequently between the input and overt behaviours that use the internal pulse as a temporal reference). Studies of externally paced finger tapping responses (the sensorimotor synchronization paradigm) have revealed that these mechanisms include reactive error correction processes entailing phase and period correction [49]. Moreover, additional online mechanisms include anticipatory processes that allow the timing of upcoming sensory events to be predicted during ongoing tempo changes [1,130]. These processes may be engaged differently depending on attentional resources [131], individual characteristics [132,133] and task demands [134,135]. Temporal adaptation and anticipation are nevertheless fundamental processes, and evidence for their operation has been observed in the macaque monkey [107]. In humans, adaptation and anticipation mechanisms are also sensitive to hierarchical metric structures, and can use multiple periodicities in the stimulus to stabilize performance [14,136].

#### (ii) Going beyond one-to-one using grouping and subdivision

Research has identified two fundamental processes that allow going beyond one-to-one mapping between internal pulses and an external periodic input. Subdivision provides an internal representation of pulses with faster periods than the input, whereas grouping provides an internal representation of pulses slower than the input period [12,49]. Both grouping and subdivision open to possible simultaneous internal representation of multiple pulses, a cornerstone of meter processing in humans [8,10]. Importantly, subdivision [8,137] and grouping [9,24] seem to be engaged spontaneously in humans. Moreover, both processes seem to be biased to binary ratios [8,9,24,138], although this may be influenced by long-term exposure [139] and input properties [140].

Despite these intrinsic biases, humans show extraordinary flexibility to employ subdivision and grouping processes based on top-down intention. This is evidenced by finger-tapping experiments [138,141], and studies capturing neural responses using the frequency-tagging approach [142–145] and contrast-based methods [53,54,146]. Furthermore, musically trained individuals show similar flexibility in controlling the phase of internal pulse representation with respect to the input, as well as the phase with which overt movement is coupled to this internal representation [8,14,147]. Such top-down flexibility is an important characteristic of human coordination abilities, which could be built upon fundamental mode-locking mechanisms proposed by neurodynamical models [148]. This human-specific top-down flexibility can be used to investigate the limits in the range of absolute pulse periods that can be internally represented, while partially controlling for limits inherent to the measured secondary process (e.g. biomechanical limits of movement). For example, grouping has been used to reveal slow limits of absolute pulse periods, whereas mental subdivision and antiphase tapping has been used to reveal fast limits (for a review, see [6,12,49,149]).

#### (iii) Excluding passive reactions to the input

A common concern with one-to-one mapping is to rule out the possibility that the system is being passively driven by the input. As discussed above, a perfectly periodic sensory input may be trivially expected to give rise to a perfectly periodic response (either neural or behavioural). Hence, a requirement for zero or negative asynchrony between the input and response has been proposed, to ensure that the response ‘predicts’ the input instead of passively reacting to it [40]. This approach can be easily applied to transiently changing signals where the phase of the input and the response can be located at specific time points of the signals based on reasonable assumptions (e.g. one-to-one finger tapping to brief metronome sounds).

However, locating the relevant time points may be difficult with smooth continuous signals [11,83,150,151]. Moreover, beyond the conventional one-to-one finger tapping paradigm, it is often not feasible to determine the target time points in the input. In spontaneous one-to-*N* tapping with a fast metronome, for example, it is often difficult to know which metronome ticks are the targets. In fact, even in one-to-one coordination, ‘predictive’ responses can be simply considered a precisely timed ‘reactive’ responses to previous events in the input [12]. Nevertheless, it is evident that most tapping behaviours described in humans and non-human primates are not simply reactive responses. When the periodic input stops, subjects can continue to tap with a very similar period (if instructed or trained appropriately). Thus, the neural representation of the pulse can be studied in a synchronization-continuation task, where initially the subjects synchronize movement to a periodic stimulus, followed by an internally driven epoch, where the neural pulse runs endogenously in the absence of the auditory input [70]. Moreover, the possibility of passive reactions can be excluded when the mapping from input to internal meter is not one-to-one.

### (b) Level 2: mapping internal metric pulse with strongly periodic input

In human music, rhythmic inputs are rarely metronomic, and hence do not allow one-to-one mapping from the stimulus to internal representation of a metric pulse. Yet, the physical structure of these rhythmic stimuli commonly contains prominent representation of particular metric pulses, and therefore, they can be classified as strongly periodic. A large number of studies have shown that human listeners spontaneously detect (or extract) prominent input periodicities and directly map the internal representation of metric pulses onto these periodicities (example provided in figure 1*b*) [32,84,89]. Hence, particular pulses prominently represented in an input strongly bias the periods and phase of the perceived meter, and thus allow the establishment of consistent temporal references across individuals [31,80,89]. The process of metric pulse extraction has been successfully captured in a number of models [7], including implementations based on rules [4,32,84] and filterbanks [102,152].

Besides humans, the ability to detect prominent periodic modulations in strongly periodic inputs and map them onto strictly periodic movement patterns has been observed in certain bird species [42,151], and in a sea lion after extensive training [153]. However, such behaviours have not been observed yet in non-human primates. As evidenced by scalp EEG recordings, human brain spontaneously internalizes prominent pulses in a strongly periodic sound input and differentially responds to unexpected sound changes depending on their alignment with the phase of these extracted pulses [56,57]. However, such differential responses were not elicited in macaque monkeys as they passively listened to the same auditory stimuli, indicating that their ability to map internal metric pulses onto sensory inputs may be restricted to the most fundamental level 1 [60].

It remains possible that non-human primates could learn to perform level-2-like mappings with appropriate training procedures (see [107]), which represents an exciting challenge for future studies. In fact, recent findings support the notion of certain level 2 abilities in monkeys. For example, electrophysiological recordings in the basal ganglia indicate that monkeys can chunk the beginning and end of isochronous and randomly timed (reactive) tapping sequences [154]. Moreover, preliminary observations of EEG β oscillations (frequency range 12–20 Hz) in macaques reveal magnitude dynamics that indicate a spontaneous grouping process by two intervals during passive listening to isochronous stimulus sequences [155]. Nevertheless, more research in the animals' natural setting is needed to document whether different species of birds, cetaceans and monkeys show spontaneous abilities to perform a level-2 mapping. The use of a similar research framework across species with the new video and audio pattern recognition algorithms using deep learning are promising avenues to shed light into this important subject.

Based on the available evidence on cross-species differences, it has been hypothesized that meter processing beyond level 1 may critically depend on robust connections between auditory and motor brain regions (electronic supplementary material, figure S6) [40,41]. Yet, these abilities may be also shaped by neural mechanisms widely shared across species, particularly by transformations of sound representation owing to basic nonlinear mechanisms along the auditory pathway. These nonlinearities further enhance particular periodicities prominently represented in the acoustic input, hence providing additional constraints on the space of metric pulses that may be possibly mapped onto the acoustic input [79,80].

In addition to the enhancement of prominent periodicities in the physical input by subcortical mechanisms, the mapping of metric pulses can be biased by similar constraints on absolute pulse periods as have been described for strictly periodic inputs [147]. These absolute period constraints have been further corroborated by studies capturing EEG responses to non-isochronous auditory rhythmic sequences [83,86]. When otherwise physically identical inputs were speeded up, the brain transformed the inputs by selectively enhancing periodicities in the frequency range where spontaneous emergence of pulse representations has been previously shown in behavioural studies [6,12,49]. Collecting finger tapping responses in the same participants confirmed functional significance of this neural transformation, as participants spontaneously changed their tapping period according to periodicities most prominent in their neural responses, but not necessarily in the physical structure of the input. The ability to extract and amplify slow periodicities from fast acoustic sequences may not only rely on processing along the auditory pathway, but also on connections to the cerebellum [156], which is critical for the extraction of precise temporal information from dynamic modulations of input features [156].

In summary, mapping inputs with prominent periodicities in their physical structure to internal metric pulses may rely on a number of neural mechanisms, including subcortical nonlinearities [79], cerebellar processing [156] and a frequency tuning curve of the involved neural audiomotor network [86].

### (c) Level 3: mapping internal metric pulse with weakly periodic input

While many musical rhythms contain prominent representation of specific pulses in their physical structure, this is not a general rule. In fact, it is quite common to encounter rhythms where no clear periodicity directly stands out [101,157,158]. These rhythmic inputs have often been treated as inducing in some ways weaker internal representation of metric pulses, or it has been assumed that metric pulses are not mapped onto such inputs at all [4,21,159–161]. Contrary to these views, humans seem capable of mapping a stable internal representation of metric pulses onto such weakly periodic inputs (example provided in figure 1*b*), as observed in spontaneous finger tapping paradigms [45,81,83,85,86,91,162].

**Figure 3.**
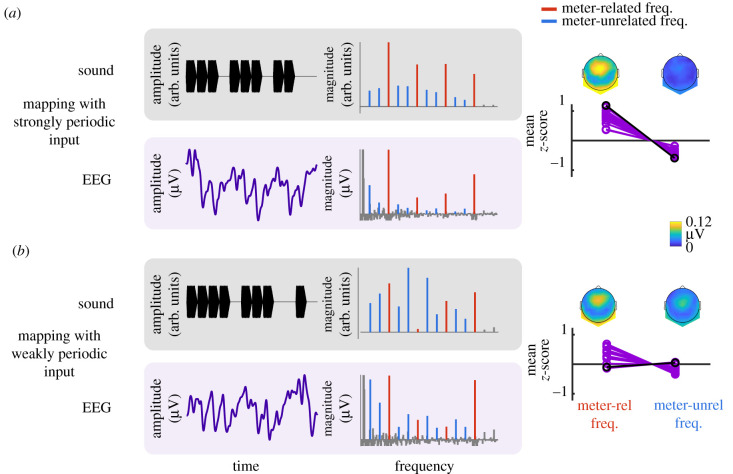
Neural transformations captured with the frequency-tagging approach. Displayed data show neural responses collected from participants as they listened to a cyclically repeated rhythmic auditory pattern without overt movement. The periods of internally mapped metric pulses were determined from spontaneous finger tapping to the same rhythmic inputs collected from the same participants in a separate session. One cycle of each rhythmic pattern is shown in black, above the corresponding EEG response (purple) (fronto-central channels, grand average across 14 participants). (*a*) Frequency-domain representations of the input (obtained using a cochlear model) and the elicited EEG show that the ‘strongly periodic input’ contained prominent representation of metric pulses already in its acoustic structure. This prominence (quantified as *z*-score) was similar in the input (black points) and the EEG responses (purple points represent individual participants), and hence can be explained by passive neural tracking of acoustic modulations. (*b*) The weakly periodic input did not contain prominent metric periodicities. However, these periodicities were selectively enhanced in the EEG responses, which cannot be easily explained by passive tracking, but rather indicate an active internal transformation.

Over the past 10 years, a growing number of studies investigated neural responses to weakly periodic inputs using EEG combined with frequency-tagging (for a review, see [29]). This approach is particularly well suited for this category of inputs because prominent representation of metric pulses in the neural responses cannot be easily confounded with passive reactions to input features. These studies provide converging evidence that the brain transforms weakly periodic inputs by selectively enhancing the representation of metric pulses (figure 3) [81,86] (for similar data obtained with magnetoencephalography, see [85]). In other words, this transformation of the input by the neural system seems to act as a sort of ‘periodization’ of the input towards the perceived metric pulses (see also [93,94,163]). This process of internal periodization is functionally relevant, as the periods of enhanced pulses closely correspond to those observed in finger-tapping responses of the same participants, when stimulated with identical acoustic inputs in a separate behavioural session [81,83,86].

Importantly, this periodization is unlikely to be explained by low-level processes in the early stages of the auditory pathway. Indeed, responses predominantly reflecting early auditory processing (i.e. brainstem auditory responses) isolated from scalp EEG signals closely track the acoustic input, hence showing little prominence of metric pulses for weakly periodic inputs [83]. This result is also corroborated by computational models of subcortical auditory processing, which provide a biologically plausible estimate of the output of neural populations at the level of the inferior colliculus in response to weakly periodic inputs [100]. By contrast, these neural representations are critically transformed at the cortical level, as reflected by significantly enhanced pulse periodicities in responses to weakly periodic inputs captured directly from Heschl's gyrus (primary auditory cortex) in human participants using intracerebral EEG [77]. Together, these results thus indicate a gradual transformation from subcortical auditory regions to higher-level regions.

Humans are capable of mapping internal metric pulses onto weakly periodic inputs, as evident in numerous musical contexts [158]. However, this mapping might be less spontaneous than mapping meter onto strongly periodic inputs (level 2), thus indicating possible engagement of different processes. This hypothesis is corroborated by tapping studies suggesting that long-term musical training may significantly improve the ability to map meter onto weakly periodic inputs [45,82]. These results suggest that differentiating strongly and weakly periodic inputs might significantly improve batteries aimed to test individual differences in meter processing [164–167]. Similarly, acknowledging the prominence of metric pulses within the physical structure of the rhythmic stimulus might lead to important insights in studies testing meter processing in non-human animals [42,151,153] or human infants [58].

Determining how exactly the brain maps a metric pulse that is not prominently represented in the physical structure of the stimulus remains an important goal for future empirical investigations. One hypothesis might be that if other (faster and/or slower) pulse periods are still prominent in the stimulus, the system could extract them and map onto internal pulses. From these, additional pulses might be bootstrapped by processes of grouping and subdivision to create a coherent metric set [91]. While slower periodicity in the stimulus can be driven by cyclic repetition of a rhythmic pattern [85,86], fast pulse period can be cued by rapid streams of events. Both of these are common in musical styles where metric pulses with intermediate periods are weakly represented in the acoustic input [101,157,158,168]. Such mechanisms have been described in neurodynamic models as mode-locking of coupled oscillators, which can reconstruct metric periodicities that are weak (or even completely absent) in the physical stimulus [91,169]. Notably, NRT has yielded promising models which posit entrained oscillation as a parsimonious explanation of behavioural and neural data across levels 1–3. These mathematical models are based on a physiologically plausible description of neural dynamics [170], yet they also rely on assumptions that still need to be demonstrated by neurophysiology. For instance, the existence of two interconnected gradient frequency networks in the human brain, necessary to model level 3 mappings, remains to be established [91,169]. In fact, recent neuroimaging work failed to observe gradient network dynamics in human premotor regions, which was predicted by previous modelling [85]. Similarly, neural dynamics observed in monkey frontal cortices might not be readily described as a resonating gradient network [36,70,121,129]. Hence, while neurodynamic models are powerful to explain data across the levels proposed in the current review, it remains to be systematically shown which parameters and parts of the network architecture are necessary to account for meter mapping at each level. A critical part of future work would be to show how these predictions correspond to the neurophysiology of different species, in order to explain why only some animals seem to achieve certain levels of the framework (as already pointed out in [34]).

### (d) Level 4: mapping internal metric pulse based on learned flexible associations

At level 3, the ability to map metric pulses onto weakly periodic inputs was conceptualized as a general skill; hence, it is expected to generalize across sensory inputs. In this sense, a skilled individual may successfully map a set of stable metric pulses onto rhythmic inputs from a particular musical tradition (e.g. music based on Afro-Cuban bembe where the sound modulations simultaneously represent pulse sets with periods in relation 2 : 2 : 3 and 2 : 3 : 2; figure 1*b*). However, while plausible, the meter mapped by this individual may be different from the meter that would be typically mapped by enculturated listeners (2 : 2 : 3 meter [168]). This illustrates that humans can use a range of indirect cues to precisely constrain the parameters of the internal metric pulses [6,101]. This may be based on learned arbitrary contextual mappings between specific rhythmic patterns and specific metric pulses, as evidenced by a recent study of musical corpora [23]. Indeed, certain rhythmic figures are often characteristic of specific genres (e.g. claves or time-line patterns in music influenced by African tradition). Hence, recognizing these figures could serve as a reliable cue to particular meters, along with numerous other cues such as particular timbres, harmonies, but also social contexts.

The associative processes at level 4 are critical to enable enculturated individuals to share temporal reference in the form of metric pulses during musical coordination behaviours, despite little direct periodic cues in the sensory input, which is characteristic for numerous musical traditions [101]. In fact, these learned mappings might even allow the listener to go ‘against’ the direct cues in the input, for instance, in ska and reggae (where a metric pulse is typically perceived in antiphase with prominent modulations in the musical stimulus). Based on the data from laboratory studies, such associative flexible mappings may be learned through multimodal exposure to musical auditory inputs accompanied by periodic vestibular stimulation resulting from active or passive movement [33,44,46]. Similarly, evidence from EEG frequency-tagging suggests that learning to move along a rhythmic input in a way that emphasizes particular metric pulses leads to enhanced representation of those pulses in the EEG activity elicited while the participants subsequently listen to the same rhythmic input without moving [43]. These results have been successfully replicated in neurodynamic models of multi-frequency networks with Hebbian plasticity, thus offering a biologically plausible mechanistic explanation [171,172]. Whether the biases towards particular meters acquired through multimodal exposure and body movement remain stable over time, to what extent they generalize (e.g. across tempi) and whether they become specifically associated with particular input features (e.g. rhythmic pattern or timbre) remains to be investigated in future studies.

## 4. Summary and perspectives

Meter has developed in *Homo sapiens* as a robust, yet highly flexible perceptual system that allows stable temporal coordination of musical behaviours among individuals, while also allowing for creativity and aesthetic complexity. Meter has been studied for a number of decades, yet the assumptions and pitfalls of different approaches used to empirically measure its internal representation are sometimes not explicitly acknowledged. The current discussion of methods that allow direct comparisons of meter representation between sensory inputs, neural activity and behavioural outputs is intended to encourage their careful use and further development. As reviewed in the current paper, these methods constitute powerful tools to study the nature of the mapping between external sensory inputs, and internal representations.

The ability to map internal metric pulse (or pulses) onto external rhythmic inputs and to use this internal representation for movement coordination has been considered a rather unitary phenomenon, exclusively present in humans and few other species of mammals and birds [40]. However, a recent hypothesis, namely the gradual audio-motor evolution (GAE) hypothesis, proposed a more continuous view according to which meter processing emerged gradually in the primate order, peaking in humans but present in simple forms across other non-human primates (for a detailed description, see [41]). Based on available empirical evidence, we have proposed a concrete framework to organize the progressively more complex forms of meter processing within four key levels. The simplest form of meter processing, here identified as level 1, is already present in monkeys, while more intricate forms of mapping between sensory inputs and internal meter seem to be fully developed in humans, supporting coordination in a flexible, culturally appropriate way, even in response to weakly periodic stimuli. GAE predicts that these human abilities evolved through a gradient of functional and structural changes within the CTBG circuit and across all the audiomotor pathways (electronic supplementary material, figure S6). Future work needs to identify the particular nature of these changes, and how they support the levels of our proposed framework. This framework might be also useful to organize previous evidence on inter-individual and developmental differences in rhythmic musical behaviours, and to integrate these findings with data from comparative studies across species (cf. [173]).

Meter processing is a phenomenon that depends on a dynamic interaction between auditory and motor systems in the brain [40,50]. In this regard, GAE suggests that the astonishing ability to perceive meter in humans is owing to their massive and dynamic flow of information between the auditory cortex, the basal ganglia and the frontal lobe (electronic supplementary material, figure S6) [41]. Critically, more complex forms of meter processing reviewed here (particularly levels 3 and 4) do not simply require a *precise* flow of information, but this information must be *transformed* to allow for a mapping from sensory inputs that are not strictly periodic onto internal periodic representation of time in the form of metric pulses [29]. Hopefully, the proposed four-level framework will guide future investigations of processes involved particularly at these more complex levels, which have received very little attention until recent years [91,101].

It can be expected that recent conceptual, methodological and analytical advances in systems neuroscience will provide novel insights into the nature of the mapping between external sensory inputs, internal representations and overt behaviour [174–176]. In particular, the access to high-density multisite recordings that allow simultaneous longitudinal recording across sensory, associative, and motor areas in behaving animals is likely to move us a critical step forward in our understanding of information processing in the central nervous system and the neural basis of cognition [177–179]. For example, an emerging view posits that the neural substrates of behaviour can be captured at the population level, considering the time-varying patterns of activity of large numbers of neurons as states of a dynamical system. Thus, the projection of neural population responses into a lower dimensional subspace defines neural trajectories that can reveal the computational strategies employed by different brain regions during cognition (figure 2) [127,128,180–182]. Similarly, the behaviour induced by the experimental task can be characterized in a space whose axes correspond to the measured behavioural variables [150]. Hence, capturing neural substrates of the behaviour relies on describing the dynamic relationship between the intrinsic neural trajectories and the behavioural state patterns [183]. In the context of this review, we hypothesize that in musical scenarios, the mapping between the dynamic neural population states representing metric pulses and behavioural states may take complex forms, particularly in humans who demonstrate flexible higher dimensional movement trajectories such as dance. Critically, it is expected that the mapping from the dynamic features of the sensory input onto neural population trajectories would be increasingly indirect when representing metric pulses from isochrony (level 1) all the way to flexible learned associations (level 4) [99]. To better understand these mappings, the analytical tools described for frequency-tagging on EEG signals can be applied to sensory inputs and the neural and behavioural state trajectories. A promising way to test these general hypotheses would involve large-scale recordings in the audiomotor system of humans and non-human animals performing tasks covering the proposed four-level framework.

In summary, the proposed conceptual framework, combined with recent advances in systems neuroscience and the methodological approaches reviewed in the current paper, may yield new empirical data to critically test and constrain mechanistic models of pulse and meter. Therefore, we hope the current review will encourage further debate in the field between proponents of different theories, using our proposed four levels as scaffolding to probe and further develop models of rhythmic behaviour.

## Endnote

1 See [30] for a live performance where Joe Dart delivers a variety of rhythmic patterns that are mapped onto the same pulse by the audience, and directly expressed by their clapping.

## Data accessibility

This article has no additional data.
